# Social stigmatization and gender inequality in education: evidence from rural Pakistan

**DOI:** 10.3389/fpsyg.2026.1840642

**Published:** 2026-10-27

**Authors:** Zawar Hussain, Cai Chenmei, Ning Zhijun, Nazia Hassan, Muhammad Iqbal

**Affiliations:** 1 School of Education, Xian Fanyi University, Xian, China; 2 School of Education, Guangzhou University, Guangzhou, China; 3 College of Teacher Education, Lingnan Normal University, Zhanjiang, China; 4 Department of Education, Allama Iqbal Open University, Islamabad, Pakistan; 5 Department of Education, Thal University, Bhakkar, Pakistan

**Keywords:** gender inequality, higher education, Pakistan, rural female, self-censorship, social stigma

## Abstract

Gender inequality in higher education remains a concern in rural Pakistan, where women’s education opportunities are shaped by social expectations concerning mobility, family responsibilities, and appropriate gender roles. This study examined how social stigmatization associated with staying at home and performing domestic roles is experienced by rural females and how public and self-stigma relate to their participation in higher education. A qualitative descriptive phenomenological design was employed. Data were collected by semi-structured interviews from 30 participants in rural Pakistan, including eight female students and nine female family members, and 13 male family and community members. Thematic analysis identified four interrelated themes: restricted mobility and chaperonage, conditional educational and career aspirations, internalized domestic identity, and protectionist concerns surrounding coeducation. The findings indicate that female education was generally valued, but support was often conditional on family approval, proximity to home, supervision, and conformity with socially accepted roles as daughters, wives, and mothers. Public stigma was reflected in shared expectations that restricted women’s mobility, educational location, and career choices. While self-stigma through self-censorship became apparent when some female students anticipated these judgments and adjusted their educational aspirations accordingly. These findings suggest that educational inequality in the setting examined is associated not only with material and institutional barriers but also with social judgments and their internalization. By applying a stigma perspective, the study provides a context-specific account of how public stigma, self-stigma, and self-censorship may interact with gendered expectations to narrow higher education opportunities for rural females.

## Introduction

Stigmatization is a term that is traced back to ancient times. The Greek word “stigma” (symbol) refers to the marks or signs that were marked on the bodies of individuals considered inferior in that society, such as criminals and enslaved people (Goffman, 2009). Stigmatization refers to the social disapproval and hostile reception of a group of people on the basis of mental, physical and lifestyle attributes that mark them as different (Jackowska, 2009). Stigmatization can block stigmatized individuals from social opportunities through discrimination or self-censorship (Corrigan, 2004). Among the social opportunities, education is crucial for achieving equality and social justice (Uduji et al., 2020). The sustainable development agenda recognizes education as a critical concern for achieving gender equality (Nimer, 2020). The 2030 Incheon Declaration and Framework for Action represented a consensus among the global education community to align with the Sustainable Development Goals (SDGs). This agenda identified that achieving gender equality through education requires an approach that not only enables males and females to access and complete the education cycle equally but also empowers them equally in and through education (Nimer, 2020; Russell, 2016; Uduji et al., 2020).

Gender inequality in education is a global phenomenon. It is evident in the different regions of the world (Klasen and Lamanna, 2008; Mezie-Okoye, 2021). The gender inequality gap is wider in low-income and male-dominated societies (Jayachandran, 2015). In Pakistan, female education receives limited social support, and reported literacy figures indicate that investment in human capital tends to favor male over females. A large share of the female population is directed towards homemaking, and prevailing norms positions men ahead of women in access to resources (Qazi and Javid, 2023). Women in Pakistan are often expected to occupy supportive domestic roles, such as preparing and serving foods for other family members. Even textbooks reinforce this supportive role of females in Pakistan (Qazi and Javid, 2023). Women have limited options for pursuing higher education, vocational, and technical degrees.

Recent research has examined the drivers of gender inequality in education, through much of it works within a limited explanatory range. A first strand attributes the gap to economic constraints. Household poverty and the direct and indirect costs of schooling reduce female enrolment, and families under financial pressure tend to prioritize the education of sons (Nartey et al., 2025; Tajammal et al., 2024). A second strand emphasizes supply-side and infrastructural barriers, including the distance to schools, the shortage of female teachers, and inadequate facilities, which are associated with lower female participant (Psaki et al., 2022). A third strand foregrounds cultural and normative constraints, such as early marriage, restricted mobility, domestic responsibilities, and son preference, that limit the schooling of girls in patriarchal settings (Ali et al., 2022; Nartey et al., 2025). Within Pakistan specifically, recent work has documented the roles of poverty, domestic work, provincial disparities, and prevailing social norms in shaping female educational outcomes (Aftab et al., 2023; Habib et al., 2024). What these accounts share is a treatment of inequality as the outcome of external barriers, whether financial, structural, or normative. Two limitations follow. First, the internal and psychological dimension of exclusion has received comparatively less attention. The reviewed studies rarely account for how the affected individuals internalize social disapproval and, through self-censorship, withdraw from educational opportunities before an external barrier is encountered (Fernández D. P. et al., 2023). Second, the cultural-norms strand describes the content of restrictive norms without a framework that connects social disapproval, hostile reception, and internalized self-concept into a single process. Stigmatization theory offers such a framework, as it describes how societal devaluation operates both through external discrimination and through the internal process of the stigmatized individuals (Corrigan, 2004; Fernández D. P. et al., 2023; Goffman, 2009) This theory has been applied widely to mental illness and health but less often to educational inequality, and, to the knowledge of the authors, its application to rural women’s higher-education experiences in Pakistan appears to remain limited in the literature review for this study. The gap addressed here is therefore not the absence of research on female education, but the absence of an account that treats gender inequality in higher education as a stigmatization process combining social disapproval with self-censorship.

Against this background, the present study uses a stigma perspective to examine how social judgments and their internalization related to rural women’s educational experiences and participation in higher education. It focuses on how expectations surrounding domestic roles, family and community responses, and anticipated social judgments. Accordingly, current study addresses the following research questions:

Q1. How do rural female participants experience social expectations and stigmatization associated with staying at home and performing domestic roles?

Q2. How is social stigmatization related to rural females’ access to and participation in higher education?

## Literature review

### Theoretical framework

Based on sociological theory of stigma this study examines the relationship between social devaluation, gendered expectations, and access to higher education. This theoretical framework brings together Goffman (1963), account of stigma, Link and Phelan (2001) conceptualization of stigma as a social process, and Corrigan (2004) distinction between public stigma, and self-stigma. Gender-role and structural perspective helps explain how expectations about women are formed and sustained, while stigma theory helps interpret how such expectations may become personally consequential when women anticipate social judgment and restrict their own educational choices.

### Stigmatization

By stigmatization, we refer to social groups that others hold adverse perceptions, stereotypes, and opinions about, or which generally exhibit comparatively low behavioral or economic performance relative to individuals in a society due to inequality against members of the social group. The sociologist Erving Goffman defines “stigma” in a pioneering study of the subject as a profoundly discrediting attribute that tends to elicit negative and sometimes hostile conduct against stigmatized individuals (Goffman, 1963). The recent studies retains this relational view while placing stigma explicitly within the study of social inequality, treating it as a driver of unequal access to resources and opportunity rather than a fixed personal trait (Addison, 2023; Tyler and Slater, 2018). Elliott et al. (1982) suggested that a stigma is a form of deviance that leads others to perceive somebody as unable to participate in an interaction. People may be deemed illegitimate interactants if they lack the ability or skills to carry on an interaction, behave erratically or inconsistently, or pose a risk to others or the interaction itself. According to Elliott et al. (1982), if a person is considered illegitimate for participation in an engagement, they are no longer protected by social norms and may be ostracized or disregarded entirely. Crocker et al. (1998) recognized the difficulties in establishing a single, defining element of stigma. However, they proposed that stigmatized persons are thought to possess “some attribute or characteristic that conveys a social identity devalued in a particular social context”. They believe that stigma originates from being part of a group or category that is viewed negatively in a particular situation. This collective identity results in negative external perceptions and social rejection of stigmatized individuals (Phelan et al., 1997). The two types of stigmas are identified by Corrigan (2004): the public stigma and self-stigma. Self-stigma refers to the internal process by which individuals internalize these negative perceptions and apply them to themselves. It operates through self-censorship, for example when a person stops seeking an opportunity such as education because of anticipated rejection of previous negative experience, and through a decline in self-esteem and a sense of diminished self-worth (Corrigan and Watson, 2002). Recent evidence continues to support this distinction between anticipated, internalized, and enacted form of stigma (Fernández D. et al., 2023). The social stigma is reinforced by the political, cultural, and economic powers. These powers act as a social mechanism that enforces cultural norms and social control over individuals who do not adhere to them. The disadvantageous consequences for stigmatized populations include social isolation, lower employment and income, poor physical and mental health, and low quality of life (Fernández D. et al., 2023; Hatzenbuehler et al., 2013). Stigma is a central component of social inequality, shaping identity formation, social inclusion, and access to services, resources, and opportunities (Link and Hatzenbuehler, 2016).

### Gender inequality in education in rural Pakistan

Education is regarded as the single most significant instrument for human progress and is increasingly acknowledged as a fundamental human right and an effective instrument for women’s empowerment (Linda, 2014). Provide girls in rural areas of Pakistan with equitable access to education, free from prejudice, remains a pressing need (Salik and Zhiyong, 2014). However, gender disparities in education remain evident in Pakistan. Although gender parity in tertiary education enrolment has improved, Pakistan continued to rank 139th in Educational Attainment in the 2024 Global Gender Gap Index, indicating persistent inequalities across educational indicators (Pal et al., 2024). The gender gap is also geographically uneven, with rural women facing particularly pronounced educational disadvantage. Census data show substantially lower literacy and educational attainment among rural women than their urban counterparts, while recent qualitative evidence from rural Pakistan identifies limited educational access, mobility restrictions, economic hardship, and sociocultural expectations as interconnected barriers to girls’ education (Kalani and Symaco, 2025; Govt. Pakistan, 2023). Gender inequality in education originates from cultural, societal, and economic causes and is more severe in rural areas, where universities are not easily accessible and students must travel considerable distance from their hometown (Kalani and Symaco, 2025). Early marriages remain common in rural areas and further reduces the likelihood of continuing to higher education, since girls in the poorest households are markedly less likely to attend school than boys (Zahra, 2024).

### Conceptual framework

Drawing the two preceding section together, the conceptual framework proposes a pathway through which social devaluation may be related to women’s participation in higher education. Stigmatization is treated as asocial condition that may operate through two related mechanisms: public stigma, expressed through family and community judgments and restrictions, and self-stigma, reflected in internalization of socially prescribed gender roles. In the rural setting examined, these processes may influence whether women view particular educational opportunities as socially acceptable or realistically available. Both mechanisms can therefore contribute to restricted access to educational opportunities, although the study does not assume that all women internalize these expectations in the same way (Figure 1).

**Figure 1:**
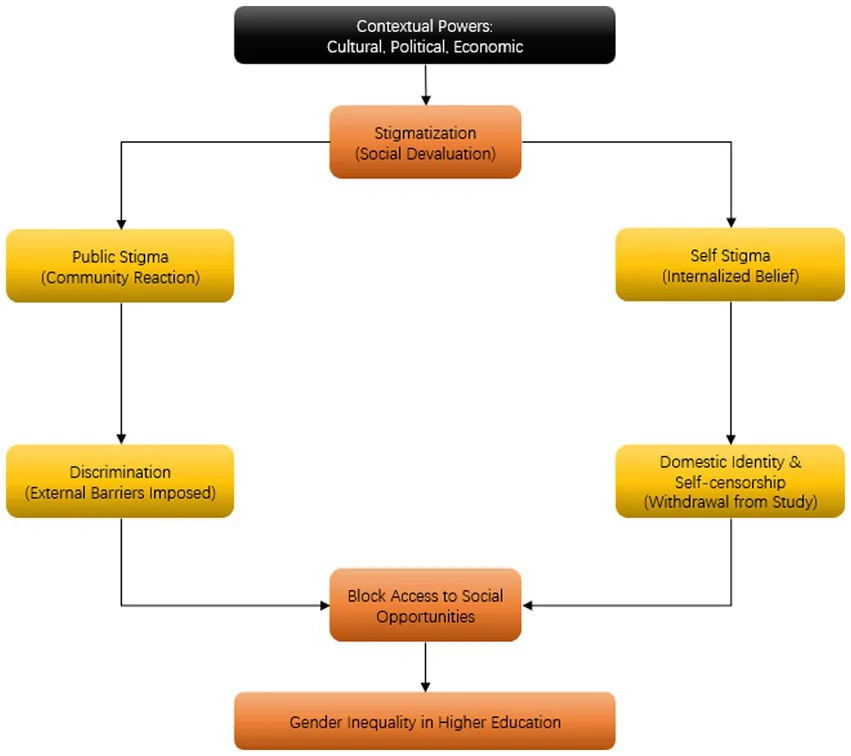
Mechanism of stigmatization to gender inequality in education.

## Methodology

### Research design

The current study adopts a qualitative approach and, more specifically, a descriptive phenomenological design to explore participants’ experience of phenomenon under examination. This design is appropriate here because the study seeks to understand how rural women and members of their families and communities experience and interpret social expectation surrounding women’s education, mobility, and domestic roles (Moustakas, 1994; Poth and Creswell, 2018). The research questions concern how stigmatization is experienced and how it is related to access to and participation in higher education, rather than the frequency of particular behaviors or the testing of a causal hypothesis. A phenomenological orientation also allows the phenomenon to be examined from the perspectives of female student as well as family and community members.

### Participants and sampling

Participants were selected through purposive sampling, because they had direct experience of or a direct role in, decisions concerning female higher education in the study area (Palinkas et al., 2015; Poth and Creswell, 2018). Recruitment of third group was extended through snowball sampling, whereby initial participants identified other community members who met the criteria. For this purpose, respondents were divided into three groups. The first group comprised eight rural female intermediate students. The second group comprised nine female family members including mothers and sisters. The third group comprised 13 male family and community members, including fathers, brothers, teachers, politicians, and journalists. The final sample therefore consisted of 30 participants.

### Data saturation

Data collection continued until saturation was reached, understood as the point at which additional interviews produced no new codes or themes relevant to the research questions (Guest et al., 2006; Hennink and Kaiser, 2022). Saturation was assessed during data collection by comparing each new interview with the developing codes and themes. In each participant group, the final interviews did not generate substantively new themes, which supported the adequacy of sample for the phenomenon examined.

### Data collection and interview procedures

Semi-structured individual interviews were conducted with the first and second participant groups, while a group interview was conducted with the male participants of the third group. The interviews were conducted by researchers who are residents of the study area and native Urdu speakers. Each interview was guides contained 14 open ended questions for students and 11 for parents and family members, with follow-up probes used to explore response in depth. Interview duration varied according to participants’ willingness to elaborate on their experience and perspective, and interviews continued until relevant domains of semi-structured interview guide had been discussed in adequate depth. The interviews were conducted in Urdu and recorded using voice-recording device. The researcher also took notes during the group interview.

### Study setting

This study was conducted in District Bhakkar, Punjab Province, Pakistan. This district has only one university, namely Thall University Bhakkar. There are 19 colleges and 1,300 primary, elementary, high, and higher secondary schools. District Bhakkar has a population of 1,647,852, of whom 51.16% are male and 48.84% are female. The district is predominantly rural, with 84.24% of its population residing in rural areas, and has a reported literacy rate of approximately 51.82% (Ahmar et al., 2022).

### Trustworthiness

Trustworthiness was addressed using the four criteria of (Lincoln and Guba, 1985): credibility, dependability, confirmability, and transferability. Credibility was supported through triangulation across the three participant groups and across two data-collection methods, individual and group interviews, so that accounts of the same phenomenon could be compared for convergence (Carter, 2014; Lincoln and Guba, 1985). Members checking was used, with participants given the opportunity to confirm that summarized interpretations reflected their intended meaning. Peer debriefing was carried out among the research team and with colleagues outside the study to examine the developing interpretations. Dependability and confirmability were supported by maintaining an audit trial documenting research decisions, coding steps, and analytic choices.

Because the researchers were familiar with study area, reflexivity was treated as an important part of research process. Their familiarity with the local social and cultural context supported the access and contextual understanding, but it also created the possibility that prior assumptions could influence interpretation. The researchers therefore used reflexive discussion and bracketing to remain attentive to their own assumptions during data collection and analysis (Moustakas, 1994). A reflexive record of the researchers’ positions, observations and impressions was kept throughout the study.

### Interview development

The interview guides were developed through a review of relevant literature, the researchers’ familiarity with study context, and discussion among the research team and other knowledgeable colleagues. The questions were designed to explore mobility, domestic responsibilities, educational experiences, career aspirations, and family decision-making, coeducation, and perceptions of appropriate female roles.

### Interview guide (Main domains)

The semi-structured guide covered the following broad domains:

1. Daily mobility, and who, if anyone, accompanies the respondent outside the home.
2. Household responsibilities and division of domestic labor.
3. Educational history and current program of study.
4. Career and further-study aspirations, and the conditions attached to them.
5. Who is involved in decisions about the respondents’ education, and mobility.
6. Views on coeducation and mixed-sex university environments.
7. Perceptions of appropriate female roles and successful women.

### Analytical procedures

The analysis was conducted within a descriptive phenomenology orientation, which directed attention to participant’s lived experiences and the meaning they attributed to education, domestic roles, mobility, and social expectations. During analysis, the researchers sought to remain close to participants’ accounts and to limit the imposition of predetermined theoretical categories. Reflexive discussion among the researchers was used to consider how prior assumptions could influence interpretation.

Data were analyzed using Braun and Clarke (2006) six-phase approach to the thematic analysis. Three researchers participated in the coding and analysis. They first read and re-read the transcript to become familiar with the data and generated initial codes inductively from participants’ accounts. Developing codes were compared and discussed collaboratively, and related codes were grouped into candidate themes. Candidate themes were reviewed against the coded extracts and the full dataset for coherence and distinctiveness. Where interpretive differences arose, the researchers returned to the relevant transcripts, discussed alternative interpretations, and reached consensus. Through this iterative process, four themes were developed: restricted mobility and chaperonage, conditional educational and career aspirations, internalized domestic identity, and protectionist narratives surrounding coeducation. The themes were developed from the data rather than predetermined by the theoretical framework. After theme development, the theoretical framework was used as an interpretive lens to examine how the identified patterns related to public stigma, self-stigma, and self-censorship.

## Findings

### Overview of themes

Four themes were developed from the data set: (1) Restricted Mobility and Chaperonage as a Condition of Access; (2) Conditional Educational and Career Aspirations: (3) Internalized Domestic Identity and the Wife-Mother-Daughter Script: and (4) Protectionist Narratives and the Case Against Coeducation. The first and third themes drew on accounts across participant groups. The second was grounded mainly in student interviews, while the fourth drew primarily on female family members and male family and community members, together with students’ accounts of supervision and mobility restrictions. The relationship among the four themes is discussed after the individual theme section.

### Theme 1: restricted mobility and chaperonage as a condition of access

Among the intermediate students, unaccompanied movement outside the home was often described as restricted rather than a matter of personal preference. Students commonly reported traveling to college with relatives or other companions and several described accompaniments as an important condition of attendance. Where a respondent occasionally traveled alone, the experience was described in term of insecurity or fear rather than independence.

*"I do not go to the market to purchase anything; our family does not allow females to go out shopping. They do not like females going to the market for any purchase."* (Respondent 1 stated)

“*When I started college, I had to go alone to the college. I was terrified of going alone. I also attended the additional coaching class. The coaching academy is within walking distance from my home. In the early days, my male teacher would often accompany me home after coaching class, as I felt very insecure.”* (Respondent 4 stated)

*“I come to college on a rickshaw with my female cousins. It has never happened that I came to the college alone. However, I feel very secure when my brother is with me to drop me off at college.”* (Respondent 5 stated)

The pattern held after college hours as well. Respondent commonly described staying inside the home once class ended, taking part in no activities beyond it, and confirmed that going out alone remained off-limits regardless of the reason.

### Theme 2: conditional educational and career aspirations

Respondents were not without ambitions; nearly all named a career they wanted for themselves. What stands out in the data is that this ambition was rarely treated as a stand-alone decision. Each respondent attached at least one of three recurring conditions to it: approval from family, the physical distance of suitable institution from home and the social acceptability of career itself for a rural woman.

*“I want to study computer science at university; if I get admitted to Thall University (the nearest university campus), it will be best for me. However, if I am unable to enroll on this campus, it will be challenging for me to study in another city. In this case, my family members will have to accompany me to a big city, or I will have to drop out of my education.”* (Respondent 1 stated)

*"I want to be a news anchor, but I know my family will not allow me to join this profession. They will not allow me to enroll in mass communication or journalism. My brother will be more resistant to joining me as a news anchor. They prefer only the teaching profession for females.”* (Respondent 3 stated)

*“I want to pursue my graduation in mathematics, and if I get enrolled in Thall University (the nearest university campus). There will be no problem for my family. However, if I need to move to another city, I have only one option: Multan, the nearest big city. I can study in Multan because my uncles live there. Other than Multan, I will not move to the big city to study."* (Respondent) 6 stated)

Distance functioned almost as a second gatekeeper alongside family approval. Proximately to a locally acceptable institution, or presence of trust male relatives in a large city, was frequently what separated a workable plan from a foreclosed one.

### Theme 3: internalized domestic identity and the wife-mother-daughter script

Beyond the practical limits on movement and career choice, respondents’ accounts pointed to something more settled: a self-understanding of womanhood built around household service and the roles of wife, mother, and daughter. Respondent 7’s account shows how mobility restrictions and domestic obligation reinforce one another with in a single account.

*“It does not matter what I am studying; I am unable to go outside alone for any activity. I come to college with my brother, and I have to perform about 80% of household activities. My mother always advises me to perform household chores, regardless of the circumstances. My family makes the decisions about my studies and my elder brother is also involved.”* (Respondent 7 stated)

This self-understanding was not confined to the students. Female family members interviewed for the study described womanhood in near-identical terms, wife, mother, daughter. And treated religious education as what equipes a woman to perform these roles well. Nearly all wanted their own daughters to eventually attend university, but hedged that wish with conditions rooted in propriety and reputation rather than in academic readiness. Respondents were also split on whether they would prioritize a sons’ or daughter higher education if forced to choose, five favored sons, four favored daughters. And even those leaning toward daughters ground the preference in the same terms: a more capable future mother, not a more independent future professional.

A similar logic appeared among male members of the community. A male teacher and social worker put in this way.

*“I believe in female education because when she becomes a mother, she can bring up her children more effectively.”* (Male teacher and social worker)

He extended the reasoning to widowhood: an educated woman, in his view, would be equipped to work and raise her children alone if she outlived her husband. Fathers and other male community members convergence on nearly the same definition of a successful female life as the female relatives did, a stable marriage, well-raised children, and conduct aligned with culture and religious expectation. Occupational or personal achievement outside that frame was rarely raised, by either group, as a marker of success.

### Theme 4: protectionist narrative and the case against coeducation

The restriction described in themes 1 and 3 were not presented by family and community members as arbitrary; they were justified through a specific, repeated narrative about risk. Father and other male community members described a female student left alone outside the home as exposed to danger romantic entanglement, harassment, or simple distraction from her studies. And treated supervision as the responsible response to that risk rather than as a restrictive requiring justification.

*“No, a female should not be sent to university education alone. It is okay if you have a good institution near you, but if not, you may want to consider moving to the city for a female university education. Moreover, if there is a coeducation, I am entirely against coeducation. It has created many problems. I have studied at university and experienced hostel life, so I am familiar with the university lifestyle. Going to university alone is not beneficial for most females. You should scur your future, and unfortunately, universities have made females realize that they are equal to men. They can do whatever they want; I have seen many females in universities jeopardizing their future. They crossed all the limits. Unfortunately, the government has created a curriculum that requires children to sit in chairs and give orders to others. If females also sit back, then who will work with their hands? If you send a female to university alone, there will be males who are not relatives. When Junaid Baghdadi and Rabia Basri, a male and a female religious and historical figure famous for their spiritual education, cannot sit and study together. Then, how can anyone else study in a co-educational setting?”* (Respondent 8 of female member stated)

Two things stand here. First the objection is not to female university education as such the respondent 8 herself completed a university degree and lived in a hostel, but to unsupervised proximity between unrelated men and women, which she frames through the religious examples of Junaid Bagdadi and Rabia Basri. Second, the same passage carried a broader anxiety about shifting gender expectations, framing greater equality between men and women at university as a danger to woman’s future rather than as an opportunity.

### How the themes related

The four themes are best understood as related rather than independent. Restricted mobility was supported by protectionist and risk-based reasoning expressed by family and community member, while students also described domestic responsibilities and future roles in term that overlapped with family expectations. Educational and career aspirations were therefore negotiated within conditions concerning family approval, proximity to an acceptable institution, supervision and the social acceptability of particular occupations. Together the themes show how external expectations and participants’ own anticipations of those expectations could shape the educational choices they considered available.

## Discussion

The findings suggest that the educational disadvantage in the setting examined was associated not simply with opposition to female education, but also with conditions attached to socially acceptable female conduct. Across the three participant groups, education was generally regarded as desirable. Support, however, often became conditional when education required independent travel, residence away from the family, coeducation, or entry into occupations considered unsuitable for women. This pattern adds nuance to recent Pakistani evidence on the enabling role of parental support. Sadaf et al. (2024) for example, found that positive parental attitudes and family support helped first-generation women enter and participate in higher education. The present accounts show a more conditional form of support in which families could value female education while also limiting travel, coeducation, educational location, and occupational choice. Support for education, therefore, did not necessarily mean unrestricted educational choice in the rural setting examined.

Restricted mobility was one of the clearest expressions of these conditions. Students described accompaniment by brothers, others male relatives, or female relative as a common condition of traveling to college. Families often framed accompaniment as protection from harassment and other perceived risks. At the same time, dependence on accompaniment narrowed the institutions and programs that student could realistically consider. The issue is therefore not adequately understood as a transport problem alone. In stigma term, independent female mobility may acquire a discrediting social meaning when it is associated with impropriety, vulnerability, or failure to conform to expected gender conduct. This finding converges with recent research identifying distance and mobility restrictions as an important barriers for women in Pakistan (Hasan et al., 2023; Rana et al., 2024). The present accounts add further detail by showing that distance was not experienced only as a geographical constraint, but also in relation to whether a women could travel alone, whether someone was available to accompany her, and whether such travel was considered socially acceptable.

The students’ career accounts reveal a related mechanism. Several participants had clear ambitions, but they evaluated those ambitions in advance against family approval, proximity to home, and the social acceptability of the occupation. A student who wished to become a news anchor, for example, already expected family resistance and recognized teaching as the profession likely to be accepted. Another anticipated abandoning higher education if admission required moving beyond locations where relatives could provide supervision. These accounts indicate that inequality operates before a formal educational decision is made. The range of imaginable choices is narrowed by anticipated reactions from significant others.

This finding helps distinguish public stigma from self-stigma in the settings examined. Public stigma was reflected in shared judgment about appropriate female conduct and in practices that restrict movement, educational location, and occupational choice. Self-stigma became relevant where socially produced expectations appeared to enter participant’s own assessment of what they could reasonably pursue. The data do not suggest that every participant accepted these expectations. Several students expressed ambitions that challenged conventional roles. The interviews instead showed a tension between aspiration and anticipated sanction, with some participants adjusting educational preferences in anticipation of family or community judgment.

The domestic construction of female identity further helps explain why support for education did not necessarily translate into equal educational opportunity. Adult women and men commonly valued female education in relation to becoming a better mother, wife, or caregiver. Education could therefore be supported partly for its contribution to family life rather than solely for women’s independent educational, occupational, or economic aspiration. Gender expectations were reported by both men and women, including mothers who supported their daughters’ education while also expressing conditions concerning location, supervision, coeducation, and future family roles. In the present study, support for education and traditional gender expectations were therefore not always opposites. Participants could support women’s education while continuing to place marriage, motherhood, caregiving and family responsibilities at the center of an appropriate female life.

Viewed through Goffman’s (1963) account of stigma and Link and Phelan’s (2001) emphasis on labeling, stereotyping, separation, status loss, and discrimination, the findings illustrate how social identity and expectations can shape access to opportunity without requiring overt exclusion in every case. Being identified primarily through the roles of daughter, wife, and mother establishes a normative standard against which independent mobility and career-oriented behavior may be judged. Corrigan and Watson (2002) distinction between public and self-stigma is useful for interpreting how collectively held judgments may become personally consequential when women anticipate them and adjust their behavior or educational choices. The contribution of this study is therefore to connect externally expressed gender expectations with anticipatory self-limitations in the specific context of rural female access to higher education.

Recent Pakistani research continues to document socio-cultural and structural barriers to women’s participation in higher education, while contemporary work on women’s empowerment also points to persistent social perception of women’s roles. The present findings add a stigma-based interpretation by drawing attention to meaning attached to female respectability, mobility, and socially legitimate roles. This perspective also connects the study to Sustainable Development Goals 4 and 5: expanding educational provision is important, but equal access may remain incomplete where social norms make use of those opportunities conditional on gendered expectations. The present findings converge with Hasan et al. (2023); and Rana et al. (2024) in identifying distance, mobility restrictions, family circumstances, and sociocultural expectations as important influences on women’s opportunities. They also nuance in two respects. First, family support for education may coexist with restrictions on travel, coeducation, educational location, and occupational choice. Second, distance and mobility may carry social meanings related to safety, respectability, and appropriate female conduct rather than functioning only as practical barriers. The stigma perspective helps interpret this additional layer by drawing attention to how anticipated social judgment may enter women’s own assessment of which educational choices are realistically available.

Taken together, the findings suggest a process rather than a list of independent barriers: gendered stereotypes define appropriate female conduct; these expectations support restrictions on mobility and decision-making; restricted choices reduce access to geographically and occupationally diverse opportunities; and anticipated social judgment can encourage self-censorship. This interpretation does not imply that rural women lack agency. Aspirations, negotiation, and selective resistance were visible in the interviews. Rather, agency was exercised within a social environment in which the perceived costs of departing from accepted gender roles are unevenly distributed. Several participations questioned conventional expectations or expressed ambitions beyond them, yet some also adjusted their plans in anticipation of family or community reactions. Their accounts suggest that negotiation and self-limitation can occur alongside aspiration rather than indicating either complete conformity or complete resistance.

### Theoretical implications

The study offers three theoretical implications for understanding stigma and gender inequality in the rural higher-education context examined. First, it illustrates how stigma theory can be applied to social evaluation of gendered conduct in higher education. In this setting, independent mobility, residence away from family, coeducation, and certain career aspirations could become socially discrediting when they conflict with expectations attached to respectable femininity.

Second, the findings connect public stigma with anticipatory self-censorship. Existing explanation of gender inequality often identifies patriarchal norms, household power, or gender-role expectations as barriers. The stigma perspective adds a mechanism through which these external expectations may become consequential before direct discrimination occurs: women anticipate how family and community members will judge particular choices and narrow those choices themselves. The study therefore does not replace gender-role or patriarchal explanations; but it refines them by identifying anticipated social devaluation as one pathway through which gender norms may influence educational behavior.

Third, the findings complicate the assumption that support for female education necessarily represents a rejection of traditional gender roles. Participants could favor education while defining its purpose in relation to motherhood, marriage, and family care. This conditional support may help explain why improvements in formal educational availability can coexist with continuing gender inequality in participation and educational choice.

### Practical implications

The findings suggest several practical areas for consideration. For provincial and national education policymakers, expanding higher education provision in underserved districts remains important, but provision should be accompanied by measures that reduce the social and practical cost of attendance. Possible measures may include reliable transport options for women, safe travel arrangements, appropriate student accommodation, and clear institutional procedures for preventing and responding to harassment.

Higher education institutions serving rural districts could strengthen outreach to families rather than treating admission as an individual student decision. Pre-admission information sessions involving parents and students could explain campus safety, transport, accommodation, academic programs, and career pathways. Institutions could also consider mentoring arrangements that connect prospective rural students and their families with current female students. Alumnae, and women professionals from similar communities.

Local education authorities, college, and community organizations could address the narrow association between female education and domestic competence. Career guidance at higher-secondary level may help students and families consider a wider range of occupations and provide practical information about scholarships, employment routs, and safe study options. Community engagement could include fathers, brothers, mothers, teachers, religious community figures, and other local actors because gender expectations in the interviews were expressed across groups rather than by men only.

Finally, interventions should avoid portraying rural families simply as opponents of the women’s education. Participants’ concerns included safety, harassment, distance, and social reputation. Policies may be credible when they address these concerns while also widening women’s educational choices. Progress toward SGD 4 and 5 therefore requires attention to both educational infrastructure and the social conditions under which rural women are permitted and feel able to use it.

## Conclusion

This study examined how social stigmatization was experienced in relation to the educational opportunities of rural female in the setting studied. Drawing on the account from female students, female family members, and male family and community members, the findings indicate that female education was generally valued, but often within boundaries shaped by expectation concerning mobility, family approval, domestic responsibilities, marriage, and motherhood. Participants commonly described women through the roles of daughter, wife, and mother, while independent travel, study away from home, coeducation, and some career choices were viewed with greater caution. These expectations shaped the conditions under which education was considered acceptable.

The findings also indicate that these social expectations may affect educational choices before a formal restriction is imposed. Female students expressed aspirations for higher education and professional career, yet their plans were often dependent on the family approval, the availability of institutions close to home, and the possibility of being accompanied or supervised. In this context, public stigma was reflected in shared judgments about appropriate female conduct, whereas self-stigma or self-censorship became relevant where women anticipated these judgments and adjusted their educational or career choices accordingly. The study therefore suggests that gender inequality in higher education in this rural setting is related not only to access to institutions, but also to the social meanings attached to female mobility, respectability, and appropriate gender roles.

Overall, the study provides a stigma-based interpretations of gender inequalities in higher education within the rural Pakistani context examined. Restrictions on mobility, conditional support for education, limited career choice, and the internalization of domestic-role expectations may work together to narrow the educational opportunities available to rural women. At the same time, the participants’ accounts show that rural female have educational and career aspirations and do not respond these expectations uniformly. Because the evidence comes from a qualitative study conducted in a single district, the conclusions should be understood as context specific rather than representation of all rural women in Pakistan. Within this setting, reducing gender inequality in higher education may require attention both to educational access and to the family and community expectations that shape whether women are able to make use of those opportunities.

## Limitations and future research directions

This study has several limitations. First, it was conducted in single rural district and involved a purposively selected group of female students, female family members, and male family and community members. The findings therefore provide a context-specific account and are not intended to represent all rural communities in Pakistan. Second, the study focused on experience related to higher education. Future research could examine whether similar processes are present at other educational levels and in rural communities across different provinces. Comparative studies may also explore how mobility, family expectations, and anticipated social judgment vary across socioeconomic and cultural settings.

## Data Availability

The raw data supporting the conclusions of this article will be made available by the authors, without undue reservation.
